# Protocol for the immediate delivery versus  expectant care of women with preterm prelabour rupture of the membranes  close to term (PPROMT) Trial [ISRCTN44485060]

**DOI:** 10.1186/1471-2393-6-9

**Published:** 2006-03-23

**Authors:** Jonathan M Morris, Christine L Roberts, Caroline A Crowther, Sarah L Buchanan, David J Henderson-Smart, Glenn Salkeld

**Affiliations:** 1 Department of Obstetrics and Gynaecology, Royal North Shore Hospital, St Leonards, NSW 2065, Australia; 2 Centre for Perinatal Health Services Research, PO Box M40, Missenden Rd NSW 2050, Australia; 3 Department of Obstetrics and Gynaecology, The University of Adelaide, Women's and Children's Hospital, 72 King William Road, Adelaide 5006, Australia; 4 School of Public Health, Edward Ford Building, University of Sydney NSW 2006, Australia

## Abstract

**Background:**

Preterm prelabour rupture of membranes (PPROM) complicates up to 2% of all pregnancies and is the cause of 40% of all preterm births. The optimal management of women with PPROM prior to 37 weeks, is not known. Furthermore, diversity in current clinical practice suggests uncertainty about the appropriate clinical management.

There are two options for managing PPROM, expectant management (a wait and see approach) or early planned birth. Infection is the main risk for women in which management is expectant. This risk need to be balanced against the risk of iatrogenic prematurity if early delivery is planned. The different treatment options may also have different health care costs. Expectant management results in prolonged antenatal hospitalisation while planned early delivery may necessitate intensive care of the neonate for problems associated with prematurity.

**Methods/Design:**

We aim to evaluate the effectiveness of early planned birth compared with expectant management for women with PPROM between 34 weeks and 36^6 ^weeks gestation, in a randomised controlled trial. A secondary aim is a cost analysis to establish the economic impact of the two treatment options and establish the treatment preferences of women with PPROM close to term.

The early planned birth group will be delivered within 24 hours according to local management protocols. In the expectant management group birth will occur after spontaneous labour, at term or when the attending clinician feels that birth is indicated according to usual care. Approximately 1812 women with PPROM at 34–36^6 ^weeks gestation will be recruited for the trial.

The primary outcome of the study is neonatal sepsis. Secondary infant outcomes include respiratory distress, perinatal mortality, neonatal intensive care unit admission, assisted ventilation and early infant development. Secondary maternal outcomes include chorioamnionitis, postpartum infection treated with antibiotics, antepartum haemorrhage, induction of labour, mode of delivery, maternal satisfaction with care, duration of hospitalisation, and maternal wellbeing at four months postpartum.

**Discussion:**

This trial will provide evidence on the optimal care for women with PPROM close to term (34–37 weeks gestation). Consideration of both the clinical and economic sequelae of the management of PPROM will enable informed decision making and guideline development.

## Background

Prelabour rupture of the membranes (rupture of the membranes prior to the onset of labour) occurs in 20% of all births and 40% of all preterm births [1-3]. When prelabour rupture of the membranes occurs **at term **there is good evidence that early delivery is associated with a lower incidence of maternal infection and increased maternal satisfaction compared with expectant management [2]. However, the optimal management of women with **preterm **prelabour rupture of membranes (PPROM) prior to 37 weeks, is not known.

### PPROM near term: a management dilemma

Following membrane rupture the preterm fetus is at risk of a number of complications such as: prematurity, placental abruption, ascending infection, intrapartum fetal distress and cord prolapse [4-6]. Abruptio placentae complicates pregnancy for 5–6% of women with PPROM [5]. As histological chorioamnionitis is more common in women with pregnancies complicated with PPROM compared with preterm or term controls [7], infection is the main risk for women in which management is expectant. These risks need to be balanced against the attendant risk of iatrogenic prematurity if early delivery is planned.

At extreme preterm gestations (less than 30 weeks), in the absence of maternal or fetal compromise, there is unanimity in that expectant management to allow further fetal maturation is desirable [8]. This is because the preterm fetus born prior to 30 weeks has increased risk of neonatal mortality, intraventricular haemorrhage, hyaline membrane disease and necrotizing enterocolitis. These risks, associated with immaturity, are reduced as the gestational age extends beyond 30 weeks [9].

At gestations nearer to term the benefit to the fetus of pregnancy prolongation following PPROM is uncertain such that by 34 weeks it has been suggested that there is no longer benefit for the fetus in the face of risks of intrauterine infection [10]. Decisions to electively deliver a fetus preterm however require grounding in good clinical evidence as mild prematurity is associated with a significant health burden [11]. On the other hand, expectant management means mothers are often hospitalised for prolonged periods with the consequent health budgetary implications.

### Fetal and neonatal risks at 34–37 weeks gestation

Clinical decision-making requires consideration of the potential risks and benefits of induction of labour against expectant management with delivery at term or when complications such as chorioamnionitis intervene necessitating delivery. The aim of such management is to maximise the benefits of fetal maturity while avoiding the potential harms of remaining in utero. At gestations between 34 and 37 weeks, whilst the neonate is potentially at increased risk of respiratory distress, difficulty with thermoregulation and difficulty with breast feeding these risks need to be balanced against the increased incidence of chorioamnionitis associated with expectant management in women with PPROM [2]. Histological evidence of chorioamnionitis is present in up to 50% of women who give birth preterm and is often not associated with clinical symptoms or signs [12]. Chorioamnionitis is a known significant risk factor for the neonate for the development of cerebral palsy [12,13]. It is possible that there are increased risks of long term adverse neurological outcomes in those infants whose mothers are managed expectantly with PPROM by increasing their duration of exposure to often subclinical chorioamnionitis.

### Systematic review of the evidence on care of PPROM <37 weeks

We have published a protocol for a Cochrane systematic review of randomised controlled trials to assess immediate delivery compared with expectant care in women with ruptured membranes between 34 and 37 weeks [14]. Our MEDLINE search used the terms (premature or preterm) and (rupture of membranes) or 'PROM' and (induction of labour) and randomised controlled trial) and identified three randomised controlled trials (outlined below) that we have included in a meta-analysis (Table 1).

**Table 1:** Meta-analysis of three trials comparing early planned delivery versus expectant management for preterm premature ruptured membranes between 34–37 weeks.

**Outcome**	**No. of Trials**	**No. of Women**	**Immediate Delivery**	**Expectant Care**	**RR (95% CI)**
Caesarean Section	2	140	8/72 (11.1%)	6/68 (8.8%)	1.21 (0.45 – 3.28)
Chorioamnionitis	3	260	8/129 (6.2%)	31/131 (23.6%)	0.25 (0.12 – 0.53)
Neonatal Sepsis	3	257	9/128 (7.0%)	8/129 (6.2%)	1.00 (0.41 – 2.45)
RDS	3	257	5/128 (3.9%)	3/129 (2.3%)	1.56 (0.41 – 5.97)
Perinatal Mortality	3	260	2/129 (1.6%)	2/131 (1.5%)	1.24 (0.19 – 8.06)

Spinnato studied 47 patients with PPROM who had documented fetal lung maturation and randomised them to immediate or delayed delivery [15]. Expectant management was associated with increased maternal sepsis but the study lacked power to detect any significant neonatal morbidity. In contrast, Mercer et al (n = 93) found a non-significant 52% increase in maternal infectious morbidity with expectant management but a similar incidence of abdominal delivery and neonatal infection [16]. Both Spinnato and Mercer required amniocentesis and biochemical suggestion of pulmonary maturity prior to being eligible in the study [15,16].

Naef et al randomised 120 women to expectant management or immediate delivery and found no clinically significant neonatal advantages to expectant management of ruptured membranes at this gestational age [17]. Additionally there was a decrease in antepartum hospitalization in those women randomized to immediate delivery. The sample size was insufficient to detect changes in sepsis or potentially clinically significant differences in respiratory distress.

Overall, there was insufficient power to detect differences in important endpoints such as neonatal sepsis, respiratory distress, newborn intensive care resource use or economic outcomes (Table 1). Although no differences in neonatal sepsis were seen, the systematic review suggests that over 1 in 20 babies exposed to PPROM at 34–37 weeks are at risk of neonatal sepsis (Table 1). Although immediate delivery was associated with a significant decrease in chorioamnionitis compared with those pregnancies that were managed expectantly, chorioamnionitis is an intermediate histological outcome and does not necessarily reflect maternal or infant morbidity. Thus there is insufficient evidence on the benefits and harms of immediate delivery compared with expectant care for women with ruptured membranes between 34 and 37 weeks gestation to make recommendations for clinical practice. Further trials are necessary that should be of high quality, assess serious maternal and infant morbidity, assess the costs of care and provide information on neurodevelopment at childhood follow up.

### Current clinical practice in the management of PPROM in Australia and New Zealand

In a recent survey of the Fellows and Members of the Royal Australian and New Zealand College of Obstetricians and Gynaecologists, we found there was no consensus on the management of PPROM [8]. In women who rupture their membranes at gestations greater than 34 weeks, 49% of obstetricians would manage these women expectantly with the patients remaining in hospital, while 51% of obstetricians would plan to deliver these women prior to term. This demonstrates the current diverse approach to the care of women with PPROM at greater than 34 weeks gestation, suggests that there is clinical equipoise and demonstrates the appropriateness of a clinical trial. Furthermore, 65% of the responding obstetricians would be willing to enrol women in a randomised controlled trial of the management of PPROM at gestations greater than 34 weeks.

### Resource and cost implications of planned early delivery and expectant management of PPROM

Expectant management may result in prolonged antenatal hospitalization, with antibiotic treatment and regular monitoring, and intensive care of the neonate in the event of sepsis. On the other hand, planned early delivery may necessitate intensive care of the neonate for problems associated with prematurity (eg respiratory distress). While some data are available for the US[18,19] and the UK[20], it is not known whether there are differences in the overall cost of care in the Australian setting. However, it is likely that the biggest drivers of health system costs will be the number of days admitted to hospital, particularly neonatal intensive care.

The trial is designed and sufficiently sized to detect a clinically significant difference in neonatal sepsis. We do not, however, expect differences in mortality or long-term morbidity between planned early delivery and expectant management. In the absence of differences in these final clinical outcomes, optimal treatment decisions will also require information about comparative resource use, and information about the preferences of women with PPROM. The secondary aim of the project is therefore to conduct a cost analysis of planned early delivery versus expectant management of PPROM and to establish the treatment preferences of women with PPROM.

Planned early delivery and expectant management represent very different experiences for the women involved. Currently, the relative importance to women of the attributes (health and non-health outcomes and process attributes) of the treatment alternatives is unknown. Discrete choice methodology takes into account the preferences of the mother and has been previously used to explore preferences in a number of relevant areas including intrapartum care[21], miscarriage[22], labour induction[23] and IVF[24]. Maternal preference is likely to assume more importance in the choice of management (of PPROM) if the long term outcomes of treatment are equivalent.

## Methods/Design

### Aims

The primary aim of the project is to conduct a randomised controlled trial to answer the clinical question: In women with preterm prelabour ruptured membranes (PPROM) between 34 weeks and 36^6 ^weeks gestation is planned early delivery compared with expectant management associated with less neonatal and maternal morbidity?

The secondary aim is to perform an economic analysis of the outcomes to establish the economic impact of planned early delivery compared with expectant management. The economic analysis will determine:

• the net impact of each intervention on hospital resources.

• which attributes of the treatment alternatives (such as time in hospital, risk of adverse outcomes, continuity of care etc) are important to women.

• the relative importance of these attributes.

• the extent to which women are prepared to make trade-offs between attributes.

### Hypotheses

The primary hypotheses are that early planned delivery of women with PPROM close to term is associated with:

1. less short-term neonatal and maternal morbidity compared with expectant management.

2. fewer economic costs compared with expectant management.

3. no difference in long term morbidity or mortality

### Study design

We will use a randomised controlled clinical trial to assess the impact of early planned birth versus expectant management.

### Setting

The study will be carried out in participating tertiary obstetric, metropolitan and rural hospitals with facilities to manage deliveries at or greater than 34 weeks.

### Participants/Eligibility criteria

Pregnant women between 34 and 36^6 ^weeks gestation with PPROM and a singleton pregnancy will be invited to participate in the trial. Women with ruptured membranes prior to 34 weeks gestation will become eligible at 34 weeks should their latency period extend to this gestation. Rupture of the membranes will be determined clinically and/or confirmed by a positive amnicator swab performed at the time of speculum examination. Gestational age will be determined by last menstrual period and/or by early ultrasound if there is a discrepancy of more than 7 days to that of the estimated date of confinement from the last menstrual period.

#### Exclusion criteria

Women who are in established labour, have clinical evidence of chorioamnionitis or other indications for immediate delivery such as meconium staining of the liquor or an antepartum haemorrhage or any other contraindication to expectant management will be excluded from the study. The presence of Group B streptococcus on urine or genital tract culture will not be a specific indication for exclusion from the study.

### Procedures, recruitment, randomisation and collection of baseline data

Eligible women will be identified by a local research co-ordinator and staff. Eligible women will be given the trial information sheet, counselled and encouraged to discuss the trial with her family. Once women have consented to be involved in the trial, their entry details will be recorded on a trial entry form and they will be randomised via a central telephone randomisation service to one of two treatment groups, early planned birth or expectant management.

The randomisation schedule will be prepared by a researcher not involved with treatment allocation. Randomisation will be 1:1, in balanced variable blocks and stratified by centre.

All women will have baseline demographic, past obstetric and medical histories recorded. Maternal temperature will be recorded at a time as close to randomisation as possible. A vaginal swab will be collected from each participating woman and a fetal heart rate tracing obtained. Antibiotic administration is regarded as best practice in women presenting with PPROM and these should be administered in accordance with the policy of the participating centre [25]. At the collaborating centres, data collection will be the responsibility of the local research coordinator.

### Intervention

#### Early planned delivery group

Those women randomised to early planned birth will have delivery scheduled as close to randomisation as possible and preferably within 24 hours of randomisation. The mode of birth will be determined by usual obstetric indications. Antibiotics should be continued in the intrapartum period.

#### Expectant management group

In women randomised to expectant management birth will occur after spontaneous labour, at term or when the attending clinician feels that birth is indicated according to usual care.

Care of the all women will be otherwise managed by the obstetric team with care of the infant by the attending neonatologist.

### Follow up of women and infants in both treatment groups

At birth arrangements should be made for the placenta to be examined histologically for chorioamnionitis and funisitis. The histological examiners will be blinded to treatment allocation. After birth, information will be obtained relating to birth and infant outcomes from the woman's and infant's case notes by the local research coordinator. A full blood count (FBC) will be collected on all babies with clinical signs consistent with bacterial, fungal or yeast infection and on all babies commenced on antibiotics. Four month questionnaires will be posted out to assess maternal wellbeing, satisfaction with care, breast feeding duration and early infant development.

### Outcome measures

#### Baseline data collection

To assess the comparability of the study groups, baseline demographic and medical information will be collected from the medical record at the time of entry into the study.

#### Primary outcome measure

The primary outcome will be the incidence of neonatal sepsis. Sepsis will be classified as either definite or probable by an adjudication committee who will be unaware of the allocation group.

**Definite systemic neonatal infection **will be defined as the presence of clinical signs of infection and a positive culture of a known pathogen from blood or cerebrospinal fluid. For organisms of low virulence and/or high likelihood of skin contamination of the blood culture, such as coagulase negative *staphylococcus*, both a positive blood culture and an abnormal full blood count (FBC) are required.

*Clinical signs of infection *include respiratory distress, apnoea, lethargy, abnormal level of consciousness circulatory compromise (including hypotension, poor perfusion, need for ionotropic support or volume expansion), poor feeding and/or temperature instability.

*An abnormal FBC count *includes an abnormal white cell count, low platelet count, abnormal neutrophil count, raised immature neutrophil count, raised immature to total neutrophil ratio and/or degenerative morphological changes to neutrophils (toxic granulation or vacuolization).

**Probable neonatal infection **will be defined as the presence of clinical signs of infection where the baby was treated with antibiotics for 5 or more days together with one or more of: an abnormal FBC; an abnormal acute phase reactant (including C reactive protein [CRP] or positive Group B Streptococcal [GBS] antigen on bladder tap urine, blood or cerebrospinal fluid); growth of a known virulent pathogen (GBS, E. coli, Listeria) from surface swabs; or a histologic diagnosis of pneumonia in an early neonatal death.

#### Secondary outcome measures

##### Secondary infant outcomes

will include respiratory distress, perinatal mortality, duration of stay in a neonatal intensive or special care unit, duration of stay in hospital, birth weight, Apgar score at 5 minutes, any assisted ventilation, early infant development as measured by the women at four months (corrected age) using the ages for stages questionnaire [26].

##### Secondary maternal outcomes

will include antepartum, intrapartum and postpartum outcomes. Antepartum outcomes include chorioamnionitis, antepartum haemorrhage, thrombosis /thromboembolism, cord prolapse, treatment with antibiotics, and health service utilisation including laboratory tests, fetal well-being investigations and duration of hospitalisation. Intrapartum outcomes include induction of labour, failed induction of labour, caesarean section, assisted vaginal delivery, intrapartum bleeding or intrapartum fever and treatment with antibiotics. Placental swabs and histology will also be collected if available. Postpartum outcomes including postpartum infection treated with antibiotics, thrombosis/thromboembolism duration of hospitalisation, and initiation of breast feeding will be obtained from hospital records. Maternal satisfaction with care, maternal wellbeing, anxiety, and postnatal depression, and duration of breast feeding will be assessed at four months postpartum using self-report questionnaires. Maternal wellbeing will be assessed using the SF36 Health Survey Questionnaire [27]. SF-36 data will be examined as a single item measure of health and also by the Physical Component Summary and the Mental Component Summary. The scores for women randomised to immediately delivery and expectant care will be compared at baseline and at 4 months postpartum. Anxiety will be measured by the state component of the short Spielberger anxiety scale which has been extensively used and validated [28]. Postnatal depression will be assessed using the 10-item Edinburgh Postnatal Depression Scale, a self-report measure of depression developed for use in the postpartum period [29].

### Economic evaluation

The economic questions being addressed in this study are "given the equivalence in long term morbidity and mortality between immediate delivery and expectant management for women with PPROM, what is the least cost alternative from the viewpoint of a health care funder"? and "what are the (utility-based) preferences of women with PPROM for their clinical management"? A cost analysis will be conducted in accordance with the methods outlined by Drummond et al 1997 [30]. This will involve an initial analysis on what resource utilization data can be collected on the patient case report forms, from patient medical records and from routine data collection. It is likely that the biggest drivers of cost will be the number of days admitted to hospital, particularly neonatal intensive care. The methodology for hospital cost will include micro-costing (where differences in resource utilization between patient groups are likely to be important to the final results) and components of case mix cost weights (where utilization is more likely to be similar within patient groups).

Discrete choice methods will be used to establish maternal preferences, using established methods [31]. We will generate a questionnaire based on pair-wise choices similar to the example shown in Table 2. The questionnaire will be administered in face-to-face interviews with a sample of women enrolled in the PPROMT trial. A deterministic sensitivity analysis will be employed where individual parameters are varied one at a time. A random effects probit regression analysis will be used to analyse patient responses to the discrete choice survey, identifying which attributes are important, and the trade-offs between attributes that women are prepared to make.

**Table 2:** Example of a discrete choice question with pair-wise choices

	**Early planned birth**	**Wait for labour**
Time in hospital prior to giving birth	No time	2 weeks
Likelihood of spontaneous vaginal birth	X%	Y%
Time mother spends in hospital after giving birth	1 week	3 weeks
Time baby spends in neonatal intensive car	2 weeks	No time
Which would you choose? (Please circle one only)	Early planned birth or Wait for labour	

### Statistical issues

#### Sample size

The trial is designed to demonstrate a 50% reduction in proven or probable neonatal sepsis from 5% in those women managed expectantly to 2.5% in those women managed with immediate delivery. This requires a total sample size of 1812 women with 80% power and a significance level of p = 0.05. The estimated neonatal sepsis rate of 5% is based on discussions with neonatologists and is conservative compared with the systematic review (6.2% in control arms, where a 50% reduction would require 1448 women). For women who agree to participate we expect almost complete follow-up on the primary outcomes as these will be obtained from the medical records. This sample size will also have adequate power to demonstrate an increase in the need for ventilatory support from 4% in the expectant arm to 7% in the immediate delivery arm.

#### Data analysis

Analyses will be by intention to treat, including withdrawals and losses to follow-up. Study groups will be compared in terms of baseline characteristics. As this is a randomised trial, we would anticipate minimal differences in baseline characteristics. If however, important differences are found, these potential confounders will be adjusted for in the analysis of outcomes. For the primary outcome, the relative risk (and 95% confidence interval) of neonatal sepsis in the early planned delivery group compared with the expectant group will be calculated. If adjustment for confounding is necessary logistic regression will be used. The secondary outcomes will be compared using chi-square tests of significance for categorical data and t-tests for continuous data. If adjustment for confounding is necessary logistic regression and multiple linear regression will be used respectively.

#### Interim analysis

An interim analysis by an independent data monitoring committee with established terms of reference will be conducted when 50% of women have been recruited. If there is proof beyond reasonable doubt that an arm of the study is either clearly indicated or contra-indicated, the trial will be stopped prematurely. A difference of at least three standard deviations in interim analysis of a major endpoint is needed to justify stopping the trial. All perinatal deaths will be reviewed by a multidisciplinary adverse events committee blinded to treatment allocation.

### Ethical considerations

To date the study has been approved by the following ethics committees: Northern Sydney and Central Coast Area Health Service for the Royal North Shore and Hornsby Kuringgai Hospitals (Ref. No. 0306-126M), University of Adelaide at the Women's & Children's Hospital (Ref. No. REC 1531/11/2006), Ramsay Health for North Shore Private Hospital (Ref. No. 0306-126M), University of Sydney Human Research Ethics Committee (Ref. No. 7988), Sydney South West Area Health Service Ethics Review Committee for the Royal Prince Alfred Hospital (Protocol No. X04-0167), Western Sydney Area Health Service for Westmead Hospital (Ref. No. HREC2005/2/4.23(2028)) and Nepean Hospital (Ref. No. HREC2005/2/4.23(2028)), Townsville Health Service District (Ref. No. 43/05).

### Confidentiality and data security

Participants in the trial will be identified by a study number only, with a master code sheet linking names with numbers being held securely and separately from the study data. To ensure that all information is secure, data records will be kept in a secure location at the Royal North Shore Hospital in Sydney and accessible only to research staff. As soon as all follow-up is completed the data records will be de-identified. De-identified data will be used for the statistical analysis and all publications will include only aggregated data.

The electronic version of the data will be maintained on a computer protected by password. All hard copy patient identifiable data and electronic backup files will be kept in locked cabinets, which are held in a locked room accessed only by security code and limited staff. Data files will be stored for 7 (seven) years after completion of the project as recommended by the National Health and Medical Research Council (NHMRC). Disposal of identifiable information will be done through the use of designated bags and/or a shredding machine.

## Discussion

### Outcomes and significance

At gestations remote from term, expectant management is appropriate to allow fetal maturation. When PPROM complicates pregnancies closer to term the risks of prematurity are lower and the risk to the infant of sepsis becomes of greater significance. This trial will provide evidence on the optimal care for women with PPROM close to term (34–37 weeks gestation). If it can be demonstrated that early planned birth in this clinical situation is associated with less maternal and neonatal morbidity this will change current practice. The findings of the study will also have significant resource implications as PPROM close to term is a frequent indication for antenatal admission. The trial allows for a detailed assessment of the costs associated with the care of the neonate from the two different care strategies. Given the null hypothesis of no differences between planned early birth and expectant management in mortality or long-term morbidity, information about preferences will allow women and their clinicians to make optimal treatment choices. Information about both preferences and costs will assist policymakers in deciding if, and how, treatment options are presented to women with PPROM. The results of the economic evaluation are thus expected to improve both clinical decision-making and the management of hospital resources. Therefore, analysis of both the clinical and economic sequelae of immediate birth as opposed to expectant management will enable informed decisions and guidelines to be formulated.

## Abbreviations

PPROM – preterm prelabour rupture of the membranes

FBC – full blood count

CRP – C reactive protein

GBS – Group B Streptococcus

## Competing interests

The author(s) declare that they have no competing interests.

## Authors' contributions

JM, SB, CR and CC were involved in the conception and design of the study. JM, SB, CR, CC, DHS, GS were all responsible for the drafting of the protocol and were involved in the development and implementation of the study. All authors have read and given final approval of the final manuscript.

## Pre-publication history

The pre-publication history for this paper can be accessed here:


